# The formation of political discussion networks

**DOI:** 10.1098/rsos.211609

**Published:** 2022-01-26

**Authors:** Marian-Gabriel Hâncean, Matjaž Perc, Adrian Gheorghiță, George G. Vega Yon, Bianca-Elena Mihăilă

**Affiliations:** ^1^ Department of Sociology, University of Bucharest, 90-92 Panduri St., Bucharest 050663, Romania; ^2^ Faculty of Natural Sciences and Mathematics, University of Maribor, Koroška cesta 160, 2000 Maribor, Slovenia; ^3^ Department of Medical Research, China Medical University Hospital, China Medical University, Taichung 404332, Taiwan; ^4^ Alma Mater Europaea, Slovenska ulica 17, 2000 Maribor, Slovenia; ^5^ Complexity Science Hub Vienna, Josefstädterstraße 39, 1080 Vienna, Austria; ^6^ Department of Political Science, National University of Political Studies and Public Administration, 30 Expoziției Blvd., Bucharest 012244, Romania; ^7^ Division of Epidemiology, Department of Internal Medicine, University of Utah, 295 Chipeta Way Salt Lake City, UT, 84132, USA

**Keywords:** political discussion networks, homophily, exponential random graph models for small networks, ergmito

## Abstract

Dialogues among politicians provide a window into political landscapes and relations among parties and nations. Existing research has focused on the outcomes of such dialogues and on the structure of social networks on which they take place. Little is known, however, about how political discussion networks form and which are the main driving forces behind their formation. We study a collection of ego-networks from 30 randomly sampled Romanian politicians to reveal fundamental processes behind the formation of political discussion networks. We show that ties in such networks tend to be strong and balanced, and that their organization is not affected by sex, age or education homophily. We use the exponential family of random graph models for small networks to assess likely closure mechanisms and possible homophily effects, but we note that further research and additional data are needed to fully understand the impact of context and political affiliations on the generalization of our findings.

## 1. Introduction

Individuals do not discern political information in isolation. Political facts and events are expounded through social interaction routines [1]. Informal networks contribute to the development of political views [2,3]. Interpersonal relationships are of sheer importance in predicting the content of political talks [4–6]. Therefore, it transpires that political behaviour may be regarded as an outcome of the social networks [7]. Differently said, networks manifest as spaces wherein informal conversation among relatives, friends and acquaintances occurs, and wherein political disputes may emerge [8]. Evidence suggests that informal discussions also breed political stances [9] while people tend to search, as conversation peers, more informed alters [10]. On top of that, interpersonal networks heavily shape political preferences and voting intentions [11], whereas informality consistently impacts upon individuals' political expertise, participation and engagement [12]. It has been also claimed that social networks are among the factors that enhance or even determine political involvement, action and socialization [6,13].

The political discussion networks tend to rather include the same set of people with whom important matters are discussed [8]. Previous studies have shown that political discussions take place among actors who share rather strong than weak ties [6,13–16]. It has been claimed that individuals mostly prefer interactions with emotionally close alters as that allows making easier predictions on how a possible political dispute or behaviour may unveil [8,17,18]. Discussing political topics with close friends and family entails time continuity even if the involved parties do not reach agreement in their views or positions [19,20]. In another vein, it should be noted that political information coming from close friends and family weights more for a recipient in comparison to that coming from acquaintances or strangers [21,22].

To date, current literature has mostly focused on the outcome of the political discussion networks [23] and only marginally addressed their formation processes. Even if it has already been emphasized that political discussions are likely to be embedded in small networks of strong ties (networks of close family members and friends), little is known yet about how and why ties form. Our paper calls into question the impact of structural effects and of actors’ attributes in the emergence process of political discussion networks. The investigation of the antecedents may prove valuable for understanding the patterning of political discussions into various network configurations. We employ a personal network research design [24] and analyse both attribute and network data measured on 30 individuals (egos, in social network analysis terminology) randomly sampled from a recently established and already rising star Romanian parliamentary political party. We measure both the relationships between egos and their political discussants (alters) as well as alter–alter ties. We assess the impact of local network configurations (closure mechanisms) and actors' similarity (homophily) upon the formation of political discussion networks. In doing so, we use an innovative statistical framework, i.e. exponential family random graph models adapted to the study of small-sized networks [25,26]. A collection of 30 personal networks of political discussion ties (politicians and their discussion partners—alters) is used to account for purely structural tendencies for tie formation (closed and open triangles) and for social selection effects (age-, sex- and education-related homophily). We provide evidence that might be useful for discerning the mechanisms responsible for the creation of the political discussion networks. Analysing the formation of political discussion networks is crucial for understanding democratic consolidation and development processes [11,27].

### 1.1. Homophily as an antecedent in forming social networks

People are susceptible to form, maintain or terminate social ties based on multi-dimensional similarity, e.g. age, race, gender etc. [28]. Most of the time, individuals act unconsciously in the process of shaping and forming their personal networks [29]. Homophily is an essential social selection effect that, in time, increases the homogeneity of social networks [30]. The existence of homophily has been reported in marriage relationships [31], in the formation of core networks [32], in the creation of friendship ties [33], within workplace relationships [34] and even in the case of meeting new people [35]. Homophily has also been detected in political discussion networks. For instance, people with similar political values tend to associate and develop ties [36], or tend to keep similar others as discussants [37]. Concomitantly, individuals holding strong political stances tend to retain in their networks alters with similar political preferences [19]. People cast about, as discussion partners, others who are similar on age, race, religion [4] or political opinions [15,38,39]. It has been pointed out that political homophily entails a multi-dimensional nature. That implies not only similarity on political identity or opinions but also encompasses similarity on political actions and involvement [40].

In our paper, we look at social selection in relation to four variables, notably: sex, age, education and the ego–alter tie duration similarity among alters (measured in years). We select these variables into our models to ensure that social influence (contagion) and social selection (homophily) are disentangled. Precisely, the sex, the age and the educational attainment of the individuals as well as the similarity of ego–alter tie duration cannot change as a result of social influence. We analyse a sample of equivalent individuals, i.e. people affiliated to the same political party. That allows for controlling at least one of the social contexts relevant for the formation of political discussion networks: formal participation in a political organization.

Various studies have suggested that, in general, males’ networks are likely to be more homogeneous on the sex dimension in comparison to those of the females [34,41,42]. A male–male differential homophily effect has been also reported for the case of discussing political topics [15]. Also, some findings claim that males are prone to belittle their spouses' knowledge level about political issues and are inclined not to consider them as political discussion partners [15,43,44]. Additionally, others suggest that females are less likely to embed in disagreeable networks than men [8]. In a different respect, some researchers have addressed the composition of personal networks from an age perspective. As an example, it has been elaborated that people are likely to discuss important matters with alters of similar age [32]. Conversely, people of more than 60 years old manifest the tendency to have among their confiders younger alters, especially their children. It has been shown that the networks of the elderly are mostly made up of strong ties, specifically, connections to family members and close friends [45,46]. Recent studies have illustrated that young people are prone to collect political information rather from their personal networks than from media [10]. According to other findings, youth's political behaviour is considerably influenced by parents, close friends and professors as long as these are embedded in their networks [21]. Interestingly, in the special case of the young people, their degree of political interest positively associates with the educational level attained by their parents [47]. Educational attainment was deemed as one of the statistically significant factors that affect the content of the core discussion networks [4,32] or the level of agreement in political discussion networks [15]. Studies have also shown that education is among the predictors that bias the structures of networks, in general, and of friendship networks, in particular [48].

### 1.2. Research hypotheses

The processes of tie formation within the political discussion networks have not been yet devoted complete attention. Multi-dimensional homophily has not been tested as an independent variable in the creation of ties. With that in mind, our paper aims to analyse the patterning of political discussion ties around individuals, by evaluating two classes of factors: pure structural effects and attribute-related effects. First, we are looking at pure structural predictors considered responsible for the self-organization of networks [25]. These factors are purely structural because network patterns may arise solely from the ongoing internal processes of the network ties. That is, the global observed network is the effect of local network processes [49,50]. Controlling for everything else, the presence of some ties (or small tie configurations) encourages other ties (or tie configurations) to come into existence. Second, we aim to learn whether actors' attributes may make a difference in the formation of ties. Unlike the pure structural factors, deemed to be endogenous as they are part of the network self-organization, the traits of the actors (relevant for social selection processes such as homophily) are considered exogenous factors. Consequently, we are investigating four types of homophily as attribute-related effects: sex, age, education and tie duration. We evaluate to what extent these four types of homophily may be critical in the formation of political discussion networks. If sex-, age- and education-related homophily have a rather straightforward meaning (i.e. sex, age and education similarity breeds connectivity), we would detail the ego–alter tie duration assortativity of alters. In this latter case, we are assessing whether the duration of the ego–alter relationships (measured in years) affects the probability of alters getting interconnected. In other words, we are interested to examine whether the similarity in interaction with the ego increases the likelihood of alters to create ties.

Theoretical and empirical evidence suggest that core networks and political discussion networks consistently overlap and are of similar size [51]. That also implies they are formed from strong ties and, consequently, are small sized [14]. Therefore, we retain, as structural factors, the following two network configurations: closed and open triangles [52]. Closed triangles have been theoretically shown to give rise to network closure [53]. This structural mechanism (the network closure), typical of strong tie-oriented social structures, is responsible for the generation of densely knitted or strongly connected network elements [54,55]. We are using closed and open triangles as a way of measuring network cohesion. Precisely, closed triangles produce network clustering whereas open triangles may be indicative for the presence of structural holes (two alters indirectly connected through a third one) [14,54]. The potential prevalence of structural holes may indicate individuals with complementary information. Thus, we hypothesize close triangles to entail a positive effect on the formation of political discussion ties (building of the evidence suggesting that political discussion networks are made up of strong ties)—Hypothesis 1. Conversely, we hypothesize open triangles to have a negative effect in the formation of political discussion ties (Hypothesis 2). In other words, we expect the prevalence of open triangles to be lower than expected by chance alone.

In addition to the structural factors, we also test for sex, age, education and tie duration-related homophily. We hypothesize a positive effect for the sex-related homophily in the patterning of political discussion networks (Hypothesis 3). We built this expectation on the existent claims in the literature suggesting the propensity of males to interact with males when discussing political topics. Referring to the assortative mixing related to age, education and tie duration, there is still considerable uncertainty regarding the impact of these predictors upon the composition of the political discussion networks. For example, some studies generally claim extant age biases in the patterning of relationships, while others, on the contrary, contend this idea when looking at networks by age categories. Despite the controversy surrounding the role of age in shaping the form of political discussion networks, we will hypothesize age as a predictor having a positive effect (Hypothesis 4). Education has been assessed as a factor for political agreement [15] or political preferences [8]. Nevertheless, little is known about the impact that educational attainment has on the tie formation. Building on the general evidence, available in the field of social network studies, suggesting the bias of education on the network patterning, we also expect a positive effect in the case of this predictor (Hypothesis 5). Supplementarily, we predict a positive effect of tie duration similitude between two alters on the formation of an alter–alter relationship (Hypothesis 6). We ground this prediction on the general claims made in the literature about the role of attribute similarity in shaping personal environments [28]. We will test Hypotheses 4, 5 and 6 by controlling for age groups, because the degree of homophily has been documented to generally vary depending on the nature of ties [32].

## 2. Material and methods

Our paper aims to analyse the patterning of political discussion relationships around individuals, by evaluating the impact of pure structural and social selection factors upon the tie formation. We employed a personal network research design [24]. Accordingly, focal individuals (egos), their social contacts (alters) and the relationships among these alters (alter–alter ties) were taken into account. Information about the network's characteristics (or network data) as well as the attributes of the actors or nodes (attribute data) were used in the process of testing the hypotheses.

In this study, our attention was focused on personal networks comprising political discussion ties. To avoid redundancy, we retained only the alter–alter tie configurations (redundancy means here that egos are connected to all the alters embedded in their networks). We explained the formation of a tie between two alters (the dependent variable) by evaluating the effect of two types of predictors (or independent variables). Namely, we observed local network patterns (i.e. small configurations embedded in the observed personal networks), closed and open triangles, as well as assortative mixing (i.e. sex, age, education and ego–alter tie duration homophily). It is relevant to mention for clarification that our collection of personal networks was made up of undirected ties (symmetric alter–alter ties). In these networks, the closed and open triangles terms refer to special configurations of triads (sub-graphs of three nodes) [52]. The closed triangle term adds one statistic to the model equal to the number of triangles in the network. A closed triangle is defined by three nodes fully connected ({(i,j), (j,k), (k,i)}). On the other hand, the open triangle term adds one network statistic to the model equalizing the number of distinct two-star configurations. That is, any structures of the form: {(i,j), (j,k)}. We may notice that the closed and open triangle terms mark configurations nested in the larger structure represented by the global network (the personal network). Referring to assortative mixing, the sex-related homophily term counts the number of edges (i,j) for which the attribute of i equals the attribute of j (uniform homophily). The age-related homophily term is computed as the sum of absolute differences between the age of i and the age of j, for all edges (i,j) in the network. The education-related homophily term counts the number of edges (i,j) for which the attribute of i equals the attribute of j. In our study, education was treated as a dichotomous variable: with or without higher education studies. The ego–alter tie duration similitude among alters is a term computed as the sum of absolute differences between the ego–alter tie duration of i and the ego–alter tie duration of j, for all edges (i,j) in the network; nodes i and j are alters embedded in the same network (they share the same ego).

Our data comprise 30 personal networks that belong to politicians affiliated to a local branch of a newly created and rising star Romanian parliamentary political party (dataset and code are available as [56]). At the moment of the data collection process (February, 2019), the political organization was only three months old. The study participants were randomly selected from a roster of 645 members (the total tally of people affiliated to a local branch situated in a large Romanian urban area). We used a stratified sampling, with a stratum based on age: 18–25 years old (group A), 26–55 years old (group B) and more than 56 years old (group C). In each stratum, we performed simple random sampling and selected 10 respondents. The interviews were administered by phone (computer-assisted telephone interviewing). A participant was eligible to the study if formally affiliated to the local political branch of interest. The identity of each study participant was anonymized to ensure privacy protection. This study received ethical approval (Decision no. 1, from 7 January 2019) from the Ethics Committee of the Department of Sociology (University of Bucharest). The conducted research was performed in accord with the provisions of the Ethics Code of the University of Bucharest. The study participants in the survey gave their informed consent to participate. Within the study population, the minimum age was 19 and the maximum, 72. The three clusters (groups A, B and C) were useful for controlling the age of the interviewees while fitting the statistical models. Stratified sampling was also deployed to avoid any potential sources of bias (e.g. over-representation of some age categories given the skewed distribution of the study population). Given the affiliation to the same political organization, the study participants were deemed equivalent at least on this dimension.

For each of the 30 study participants (egos), we collected data about sex (female or male), age and education (with or without higher education studies). We elicited the alters of each ego using the following name generator: *Please, mention five people with whom you discuss most often political issues*. We applied alter interpreters (questions about alters) referring to sex (female or male), age and education (with or without higher education studies). Also, we collected information about the alter–alter ties to build the personal networks. Generally, in network studies, respondents (egos) have a limited ability to report about the ties between the alters they have [24]. Therefore, we decided to administer a simple question and asked each respondent whether the alters knew each other (*Would you say that alter X and alter Y know each other?*). Given the form of the question, we elicited undirected alter–alter ties.

We profiled the people with whom egos declared to discuss most often political topics. Namely, we measured: (i) the perceived level of political agreement between ego and each of the alters (rated from 1—minimum to 5—maximum), (ii) whether ego and alters share membership to the same political party, (iii) the perceived emotional closeness between each pair of alters and between ego and alters—each ego was asked to rate the emotional closeness from 1 (minimum) to 5 (maximum), (iv) the status of the alters—family or non-family (friends or acquaintances), and (v) the ego–alter personal history—the estimated duration in years of each ego–alter tie.

We used ergmito R package to test the research hypotheses. The analysis consisted of two steps. First, we built a baseline model that only included network structural effects. We fitted three different models with the following structural terms: edge count (edges)—that accounts for the overall density of the networks, closed triangle—that accounts for closure (A-B-C, A-C; ‘my friends are friends'), open triangles—a statistic referring to all open triads in the networks (A-B-C; ‘my friends do not share a tie’). In the second step of the analysis, we assessed the role of several homophily effects in the political discussion networks. We used as a baseline model, the structural model with the overall best fit (that was identified during the first step of the analysis). More, we introduced the following terms into the models: sex-related homophily (the number of ties in which both nodes have the same sex; either males, or females), age-related homophily (the preference of an individual to interact with a peer of similar age), education-related homophily (the number of ties in which both nodes have the same educational class; either higher education studies or not) [52] and ego–alter tie duration homophily (the preference of two individuals, who have a similar history of interactions with the ego (in years), to interconnect). In all models, we controlled for the study participants' age group (with group A as the reference category). Table 1 displays the effects introduced into the statistical models. Supplementarily, we would stress that the study of the complex processes giving rise to observed configurations of relationships was limited, until recently, to medium and large size social networks (from dozens to millions of nodes). A recent advancement in the social network statistical modelling has extended the application of exponential random graph models (ERGMs) [25,57] to small-sized networks [26]. Respectively, the estimation of pooled ERGMs for small networks using maximum-likelihood estimation (MLE) is currently available with the ‘ergmito’ R package [58]. Using MLE provides greater flexibility by accelerating the estimation process—e.g. estimating bootstrapped standard errors—and allowing to test for more complex hypothesis, including adding fixed effects and interaction effects to the model; all of which we used in our analyses. Given the size of each personal network (five nodes), we modelled the observed configurations of political discussion ties using ergmito R package. Our model testing was conducted on a total of 29 networks (we only had one missing case: one of the participants did not elicit network data).

**Table 1.** Model parameter description.

effect	configuration	definition
*pure structural effects*		
edges	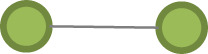	this term adds one network statistic to the model equal to the number of edges in the network
closed triangle	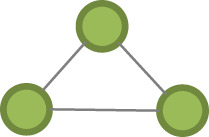	this term adds one network statistic to the model equal to the number of triangles in the network. A triangle is any set of {(i,j), (j,k), (k,i)} of three edges.
open triangle	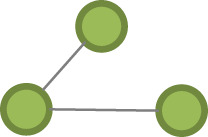	this term adds one network statistic to the model for each element in kstar(2)
*actor attribute effects*		
sex homophily	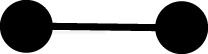	this term adds one network statistic to the model which counts the number of edges (i,j) for which sex of i equals sex of j
age homophily	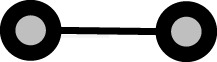	this terms add one network statistic to the model equal to the sum of absolute (age of i − age of j) for all edges (i,j) in the network, i.e. the sum of absolute differences in age
education homophily	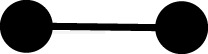	this term adds one network statistic to the model which counts the number of edges (i,j) for which education of i equals education of j
ego–alter tie duration homophily	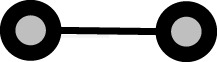	this terms add one network statistic to the model equal to the sum of absolute (duration in years of the [k–I tie] − duration in years of the [k–j]) for all edges (i,j) in the network, where k is the ego, i.e. the sum of absolute differences in the tie durations with the ego for all (i,j) edges in the network

## 3. Results

**Figure 1.**
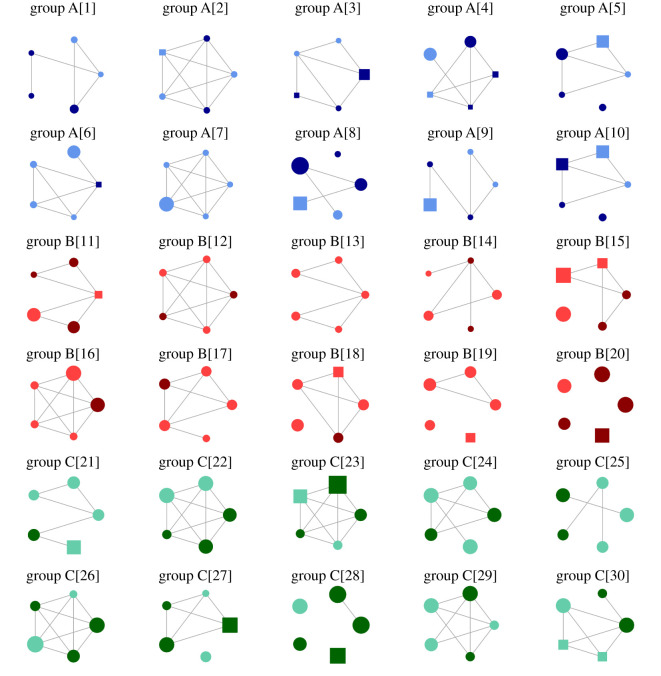
Political discussion personal networks. Colours mark classes of networks clustered on the age of study participants (blue for the 18- to 25-year-old—group A, red for the 26- to 55-year-old—group B, and green for the 56-year-old and plus—group C). Each network is indexed by its corresponding group. Dark colours illustrate females. The size of the nodes is proportional to age. Circles mark individuals with higher education studies, while squares, with less than higher education. Networks are displayed by circle layouts. The overall visualization efficiency was increased by removing the ego–alter ties, due to their redundancy. Visualizations are ordered by groups and numbered from 1 to 30.

The observed political discussion personal networks display various alter–alter tie configurations (figure 1). To avoid redundancy and render efficient visualizations, we deleted the ego–alter ties. We deployed a circle layout to emphasize the density variations from one network to another. Visual variables represent the sex, education and age of the alters within each personal network. Node size is proportional to age, dark colours mark females, while node shapes indicate educational attainment (circles mark higher education studies, squares, less than higher education studies).

Table 2 informs about the distribution of egos and alters on sex, age and education. Within the entire sample, the average age is 41 years old (s.d. = 17.2), the share of females is 0.4 and the share of respondents with higher education studies is 0.8. The age of egos correlates with the average age of their contacts (*r*_27_ = 0.83, *p* = 0.000, 95% CI [0.66, 0.92]). We performed pairwise comparisons using Wilcoxon rank sum test to examine the differences in the alters' age based on the age groups of egos (group A: 18–25 years old, group B: 26–55 years old, group C: greater than 55 years old). We observed significant differences between group A and group C (*p* < 0.001) and between group B and group C (*p* < 0.001). This indicates the alters of the elderly participants are, on average, older than the alters of the young and adult participants. Kruskal–Wallis Test was also conducted to examine the differences in the alters' sex according to the sex of the egos. A significant difference (χ^2^ = 4.683, d.f. = 1, *p* < 0.05) was found between ego males and ego females. This suggests that egos tend to have in their networks alters of similar sex. Additionally, egos with higher education studies tend to interact with alters who also have higher education studies (Kruskal–Wallis Test, χ^2^ = 5.825, d.f. = 1, *p* < 0.05).

**Table 2.** Descriptive statistics for the attributes of egos and alters. Standard deviations are provided in the parentheses. The statistics reported in the groups block refer to age averages, shares of females and of alters with higher education. Study participants are clustered in age-based groups: 18–25 years old (group A), 26–55 (group B) and greater than 55 (group C).

network	age group	ego's sex (female = 1)	share of female alters	ego's age	average age of alters	ego's education (higher education = 1)	share of alters with higher education
1	A	1	0.6	22	25.4 (5.6)	1	1.0
2	A	0	0.4	23	24.0 (0.0)	1	0.8
3	A	1	0.6	23	26.0 (9.6)	0	0.6
4	A	0	0.6	24	34.2 (17.0)	1	0.4
5	A	1	0.6	24	36.2 (13.2)	1	0.8
6	A	0	0.2	25	31.8 (13.3)	0	0.8
7	A	0	0.0	25	33.0 (17.3)	1	1.0
8	A	1	0.6	25	53.6 (19.7)	1	0.8
9	A	0	0.4	25	29.6 (13.6)	1	0.8
10	A	1	0.6	25	37.4 (13.2)	1	0.6
11	B	1	0.6	27	41.2 (13.6)	1	0.8
12	B	0	0.4	29	28.8 (0.4)	1	1.0
13	B	1	0.0	30	31.4 (3.6)	1	1.0
14	B	0	0.4	31	30.2 (8.5)	1	1.0
15	B	1	0.4	33	50.2 (14.6)	1	0.6
16	B	0	0.2	34	46.4 (17.1)	1	1.0
17	B	0	0.2	43	40.8 (6.2)	1	1.0
18	B	0	0.2	46	45.4 (4.1)	1	0.8
19	B	0	0.0	51	46.2 (5.4)	1	0.8
20	C	0	0.2	58	51.8 (5.2)	1	0.8
21	C	0	0.6	60	59.2 (11.1)	1	1.0
22	C	0	0.6	61	56.4 (17.1)	0	0.6
23	C	1	0.4	61	61.6 (4.1)	1	1.0
24	C	0	0.4	62	54.4 (6.2)	1	1.0
25	C	0	0.6	64	55.8 (16.6)	1	1.0
26	C	0	0.6	64	49.6 (16.1)	0	0.8
27	C	1	0.8	64	71.4 (7.1)	1	0.8
28	C	0	0.4	65	54.0 (13.2)	1	1.0
29	C	0	0.4	65	50.6 (13.6)	0	0.6
*groups*							
group A	*n* = 10	0.50	0.4	24.1 (1.1)	33.1 (14.7)	0.8	0.8
group B	*n* = 9	0.33	0.3	36.0 (8.5)	40.1 (11.7)	1.0	0.9
group C	*n* = 10	0.20	0.5	62.4 (2.4)	56.5 (12.5)	0.7	0.9
all network sample	*n* = 29	0.35	0.4	41.0 (17.2)	43.3 (16.3)	0.8	0.9

Table 3 provides network descriptive statistics on the ego–alter and alter–alter relationships. First, the observed personal networks display high levels of density both within the sample (*M* = 0.7, s.d. = 0.2) and across the age groups. Further, the political agreement between egos and their corresponding alters is, on average, rather high (*M* = 4.5, s.d. = 0.4), with no significant statistical variations across the age groups. Looking at the ego–alter characteristics, we notice that only 8% of all alters share the same political party affiliation as the ego. That provides grounds for our assuming that the observed ego-networks are independent. Also, 40% of all alters display a kinship relationship with the study participants. Moreover, the average duration of an ego–alter tie is 17.7 years old (s.d. = 10.5). In terms of the emotional closeness, participants tend to perceive themselves as more emotionally attached to their corresponding alters (*M* = 4.5, s.d. = 0.5) than they perceive the alter–alter emotional closeness (*M* = 2.2, s.d. = 1.3). Kruskal–Wallis test was conducted to examine the differences in the ego–alter tie duration according to the participants' age group. A significant difference (χ^2^ = 18.842, d.f. = 2, *p* < 0.05) was found among the age groups. Consequently, we performed pairwise comparisons using Wilcoxon rank sum test to examine the differences in the ego–alter tie duration based on the age groups of egos (group A: 18–25 years old, group B: 26–55 years old, group C: greater than 55 years old). We found significant differences between group A and group C (*p* < 0.001) and between group B and group C (*p* < 0.001). This result suggests that the edges embedded in the elderly's networks have a higher time duration in comparison to the ties embedded in the other networks. It also implies that political discussion networks tend to be preserved in time. Nevertheless, a longitudinal study needs to be performed in the future to properly assess this observation. On the other hand, the average duration of the ego–alter ties observed across the age groups may indicate the presence of strong ties (group A: *M* = 9.1, s.d. = 2.8; group B: *M* = 14.0, s.d. = 7.1; group C: *M* = 29.6, s.d. = 7.1).

**Table 3.** Network descriptive statistics. All personal networks have five nodes. Standard deviations are reported in parentheses. For ego–alter and alter–alter variables ((4)–(9)), we report either means or shares. In the groups block, study participants are clustered in age-based groups: 18–25 years old (group A), 26–55 (group B) and greater than 55 (group C).

network	age group	network density	political agreement	affiliation to the party	alter–alter em. closeness	family member	ego–alter tie duration (years)	ego–alter emotional closeness
(1)	(2)	(3)	(4)	(5)	(6)	(7)	(8)	(9)
1	A	0.5	5.0	0.0	2.8	0.0	3.8	3.5
2	A	1.0	3.8	0.0	4.4	0.0	6.4	4.4
3	A	0.7	4.4	0.0	3.8	0.0	8.0	3.8
4	A	0.7	4.6	0.2	2.0	0.4	11.2	5.0
5	A	0.5	4.8	0.0	1.6	0.4	12.0	4.5
6	A	0.7	4.8	0.0	3.8	0.2	9.0	4.8
7	A	1.0	4.0	0.0	3.0	0.2	6.6	5.0
8	A	0.3	4.6	0.2	0.4	0.6	11.6	4.4
9	A	0.5	3.6	0.0	2.6	0.4	11.2	4.6
10	A	0.5	4.6	0.0	2.0	0.4	11.2	5.0
11	B	0.6	4.6	0.0	0.0	0.6	14.4	5.0
12	B	1.0	4.4	0.2	0.8	0.8	12.6	4.8
13	B	0.6	4.2	0.4	3.2	0.2	6.8	4.2
14	B	0.5	5.0	0.2	2.2	0.4	4.8	4.2
15	B	0.5	4.0	0.0	1.0	0.6	19.6	5.0
16	B	1.0	4.6	0.0	2.0	0.8	28.4	5.0
17	B	0.7	4.6	0.4	3.6	0.0	10.0	3.6
18	B	0.6	4.2	0.2	2.0	0.2	16.2	3.8
19	B	0.3	4.2	0.2	3.6	0.0	12.8	4.5
20	C	0.5	4.2	0.4	0.8	0.4	21.8	4.7
21	C	1.0	5.0	0.0	2.0	0.6	30.4	5.0
22	C	1.0	5.0	0.0	0.0	1.0	40.6	5.0
23	C	0.7	4.8	0.0	3.6	0.2	27.4	4.6
24	C	0.3	5.0	0.0	5.0	0.0	27.6	5.0
25	C	1.0	5.0	0.0	0.8	0.6	34.6	4.8
26	C	0.6	4.0	0.0	0.8	0.8	35.0	4.8
27	C	0.1	4.2	0.0	2.2	0.4	26.2	4.2
28	C	0.8	3.8	0.0	1.4	0.6	35.4	4.4
29	C	0.7	5.0	0.0	1.8	0.4	16.6	3.8
*groups* mean (s.d.)								
group A		0.6 (0.2)	4.4 (0.5)	0.04 (0.08)	2.6 (1.2)	0.3 (0.2)	9.1 (2.8)	4.5 (0.5)
group B		0.6 (0.2)	4.4 (0.3)	0.18 (0.16)	2.0 (1.3)	0.4 (0.3)	14.0 (7.1)	4.5 (0.5)
group C		0.7 (0.3)	4.6 (0.5)	0.04 (0.13)	1.8 (1.5)	0.5 (0.3)	29.6 (7.1)	4.6 (0.4)
all network sample		0.7 (0.2)	4.5 (0.4)	0.08 (0.13)	2.2 (1.3)	0.4 (0.3)	17.7 (10.5)	4.5 (0.5)

In table 4, we report the results of our statistical tests. The first class of statistical models includes only the assessment of the structural effects (Models 1–3). The closed triangle term is statistically significant and positive both when evaluated alone (Model 1: Est. = 1.17, s.e. = 0.14, *p* < 0.001) or in association with the open triangle term (Model 2: Est. = 2.53, s.e. = 0.43, *p* < 0.001). On the other hand, the open triangle term holds a positive statistically significant effect only in the absence of the closed triangle factor (Model 2: Est. = 0.58, s.e. = 0.08, *p* < 0.001). In Model 3, in the presence of the closed triangle term, the prevalence of open triangles is less than expected by chance alone (Est. = −0.86, s.e. = 0.26, *p* < 0.01). This marks that the personal networks are highly cohesive, and consequently, the political discussions are embedded in full connected triads. Model 3 has the best overall fit (AIC = 319.98, BIC = 338.33). This model was retained as the structural baseline model for the subsequent model specifications (Models 4–7).

**Table 4.** Estimates of structural and homophily effects for small political discussion personal networks (ergmito models). The table also includes goodness-of-fit (GOF) statistics, number of networks used, elapsed time to fit the models. Models (1–3) only evaluate pure structural effects. Models (4–8) evaluate both structural and homophily effects. All models control for the network age class (group A: 18–25 years old is the reference category). The estimates are assigned, in parentheses, their corresponding standard error (s.e.). Significance levels are given for ***p < 0.001, **p < 0.01 and *p < 0.05.

	structural effects			structural and homophily effects				
	(1)	(2)	(3)	(4)	(5)	(6)	(7)	(8)
edges	−0.86***	−1.54***	0.67	0.70	0.85	0.55	1.16*	1.14
	(0.20)	(0.30)	(0.58)	(0.59)	(0.60)	(0.59)	(0.59)	(0.62)
closed triangle	1.17***		2.53***	2.53***	2.52***	2.53***	2.46***	2.46***
	(0.14)		(0.43)	(0.43)	(0.43)	(0.43)	(0.43)	(0.43)
open triangles		0.58***	−0.86**	−0.86**	−0.86**	−0.86**	−0.84**	−0.83**
		(0.08)	(0.26)	(0.26)	(0.26)	(0.26)	(0.26)	(0.26)
sex homophily				−0.06				−0.04
				(0.25)				(0.27)
age homophily					−0.01			0.00
					(0.01)			(0.01)
education homophily						0.21		−0.04
						(0.22)		(0.26)
ego–alter tie duration homophily							−0.04***	−0.04***
							(0.01)	(0.01)
edges * (group B: 26–55 years old)	0.01	0.01	0.01	0.01	−0.04	−0.03	−0.07	−0.04
	(0.16)	(0.18)	(0.18)	(0.18)	(0.19)	(0.19)	(0.20)	(0.21)
edges * (group C: >55 years old)	0.04	0.05	0.05	0.04	0.04	0.02	0.41	0.44
	(0.16)	(0.17)	(0.18)	(0.18)	(0.18)	(0.18)	(0.22)	(0.24)
AIC	328.33	348.98	319.98	321.92	320.29	321.13	305.55	311.27
BIC	343.01	363.66	338.33	343.94	342.31	343.15	327.57	344.30
log likelihood	−160.16	−170.49	−154.99	−154.96	−154.14	−154.56	−146.77	−146.63
no. networks	29	29	29	29	29	29	29	29
time (seconds)	0.33	0.35	0.41	0.86	2.71	0.73	2.52	4.80

The second class of ergmito models renders the evaluation of the homophily effects, controlling for closed and open triangles (Models 4–8). According to table 4, we found no evidence that sex-, age- and education-related homophily make a statistically significant impact upon the political discussion networks (*p* > 0.05). However, the ego–alter tie duration homophily displays the same statistically significant and negative estimate across all models (Est. = −0.04, s.e. = 0.01, *p* < 0.01). The negative sign of the effect indicates the presence of the homophily, i.e. the direction of the sign is affected by the incorporated computation formula—summation over absolute differences. As the absolute differences decrease, the probability of a tie increases. Hence, actors similar in their personal interaction record with the ego (measured as years) tend to interconnect more often than by chance alone (*p* < 0.01). Also, table 4 showcases that Model 7 has the best overall fit (AIC = 305.55, BIC = 327.57). In all models (1–8), we controlled for the study participants' age group (neither of the estimates were statistically significant, *p* > 0.05).

Post-estimation goodness-of-fit diagnostics (GOF) corresponding to Models 3 and 7 (the models with the best overall fit) showed the observed sufficient statistics to fall within confident intervals for most of the networks. The GOF visualizations illustrate [56]: the distribution of the sufficient statistics (edges, closed and open triangles, edges x age group B networks, edges x age group C networks, ego–alter tie duration homophily) under the fitted model (95% exact confidence intervals) versus the observed set of sufficient statistics. With only two exceptions (Model 7, ego–alter tie duration homophily), the confidence intervals generated by the models were able to cover all the networks and term combinations.

## 4. Discussion

In this paper, we aimed to discern some of the mechanisms responsible for the creation of political discussion networks. We analysed the patterning of political discussion ties around politicians by evaluating pure structural effects (closed and open triangles) and attribute-related effects (sex, age, education and ego–alter tie durations). We start this section by briefly summarizing the key results of the study. The ego–alter relationships are homogeneous on various dimensions. The age of the egos correlates with the average age of their political discussants. Also, egos tend to interact with alters of a similar sex and education. These results are consistent with the previous studies on human homophily [28] and with the work that unveiled the impact of age and education on the configuration of strong ties in core networks [32]. Additionally, our study participants perceive themselves as being emotionally close to the elicited alters and report high levels of political agreement. This is in line with previous reports gauging similar features [51,59]. Across the networks, we notice long-lasting ego–alter relationships and a consistent share of family members embedded in the study participants’ networks. The prevalence of kinship ties in the political discussion networks is, however, not unusual [13,51].

Furthermore, our data suggest that political discussions are embedded in closed triads (acceptance of Hypothesis 1) and not in open triangles (acceptance of Hypothesis 2). Sex-, age- and education-related homophily do not have a significant impact upon the formation of the observed ties (rejection of Hypotheses 3–5). However, alters who are similar in their interaction history with the ego (time duration) are more likely to develop political discussion ties (acceptance of Hypothesis 6). These findings are in line with previous work arguing that ties between individuals holding similar views are prone to be strong and balanced [36,37]. Conversely, our results de-emphasize the role of sex, age and educational level similarity in patterning the relationship configurations.

In this paper, we did not look at networks as predictors for political behaviour [2] but at processes responsible for the formation of structural configurations. Increasing the understanding of how political discussion networks form is essential for bridging the micro–macro divide as well as for the comprehension of the political world. The interpretation of our results should be context-oriented. Our analysis is focused on a special class of individuals, i.e. people engaged in the political activities associated with a formal political organization. Spatial layouts need to be brought into discussion due to their property of acting as drivers for interaction. As already highlighted [60], the composition of political discussion networks is heavily affected by the specificity of social contexts embedding individuals (e.g. workplace, public and private spaces, etc.). From this perspective, our study captures the patterning of political discussion ties around politically active individuals (politicians). Interestingly, in these networks, political discussants, even if perceived as sharing the same political views, are not affiliated to the egos' political organization. This may be an artefact of the age of the political organization (at the moment of data collection, the political party was three months old). Contrary to our initial expectations, variables such as age, sex and education were found to be not relevant for understanding the formation of the political discussion ego-networks. On the other hand, we did find support for the embeddedness of political discussions in strong and balanced triads. This evidence may indicate that people intensely involved in politics are rooted in densely connected clusters of strong ties.

The investigation of political discussion networks by employing personal network research designs and name generators is already an established methodological approach. That allows for the incorporation of structural characteristics, the disentangling of social influence and social selection, and separating context from network. Recently, available statistical frameworks for the modelling of small networks (such as ergmito) aptly allow the inspection of the formation process of political discussion networks. However, all these come with the cost of some inherent pitfalls. First, the networks are solely the result of egos’ perceptions. Studies gauging the differences between the ‘cognitive’ political discussion network, as elicited by the ego, and the ‘actual’ ego–alter and alter–alter political discussions are extremely rare. Future research work on this stream may prove fruitful in assessing the reliability of the network measurements. Second, we employed a name generator without any specific prompts [61,62]. Given our research objectives and the equivalence degree of our study participants, we estimated, in a face-validity fashion, that this type of name generator will not impose considerable measurement validity problems. Nevertheless, in the future, we intend to explore whether name generators with specific prompts give rise to political discussion networks that display different formation processes.

Discussing about the generalization of our results as well as the comparability to other similar studies, we advance at least two caveats. First, the particular profile of our study participants (members of the same political party) increases their equivalence [15] and, inversely, decrease the room for comparability to other studies [63]. Second, the surmised specificity of the Romanian political context is expected to potentially affect comparisons to previous studies performed on different national political landscapes [64]. For brevity, we note that further research and additional data are needed to fully understand the impact of context and political affiliations on the generalization of our findings.

Despite the aforementioned limits, our paper provides insights concerning the formation process of the political discussion networks. And, referring to the application of statistical models to networks in small groups [65]. First, we apply an innovative statistical framework (ergmito) specially adjusted to the study of small-sized networks [26]. Second, we give support to the idea that political discussion networks are homogeneous, with egos being surrounded by alters sharing, on average, similar traits (sex, age and education attainment). Third, we suggest that political ties are embedded in complete triangles of strong ties, wherein the history of ego–alter relationship is a predictor for the alter–alter interactions. Moreover, evidence for sex-, age- and education-related homophily was not detected in the organization of political discussion personal networks. We hope our work may also contribute to other related lines of inquiry such as those devoted to ascertaining the formation of political networks, in general, and of political elite networks, in particular.

## Data accessibility

Data and code can be accessed through the Harvard Dataverse repository for research data at: https://doi.org/10.7910/DVN/SS2GUH [56].
